# Supplementary material for: Theoretical Evaluation of Potential Cytotoxicity of Graphene Quantum Dot to Adsorbed DNA
Source: Materials (Basel). 2022 Oct 23;15(21):7435. doi: 10.3390/ma15217435 (PMC9654448; doi:10.3390/ma15217435)
Supplement: Supplementary file 1 [file materials-15-07435-s001.zip › materials-1975961-supplementary.pdf]

Support Information

Theoretical Evaluation of Potential Cytotoxicity of Graphene Quantum Dot to Adsorbed DNA

Lijun Liang [1,2,†], Xin Shen [3,4,†], Mengdi Zhou [3], Yijian Chen [3,4], Xudong Lu [3,4], Li Zhang [5,*], Wei Wang [6,*] and Jia-Wei Shen [3,4,*]

1 Center for X-Mechanics, Key Laboratory of Soft Machines and Smart Devices of Zhejiang Province, School of Aeronautics and Astronautics, Zhejiang University, Hangzhou 310027, China
2 College of Automation, Hangzhou Dianzi University, Hangzhou 310018, China
3 School of Pharmacy, Hangzhou Normal University, Hangzhou 311121, China
4 Key Laboratory of Elemene Class Anti-Cancer Chinese Medicines, Engineering Laboratory of Development and Application of Traditional Chinese Medicines, Collaborative Innovation Center of Traditional Chinese Medicines of Zhejiang Province, Hangzhou Normal University, Hangzhou 311121, China
5 Key Laboratory of Surface & Interface Science of Polymer Materials of Zhejiang Province, Department of Chemistry, Zhejiang Sci-Tech University, Hangzhou 310018, China
6 Department of Pharmacy, Hangzhou Third People's Hospital, Affiliated Hangzhou Dermatology Hospital, Zhejiang University School of Medicine, West Lake Road 38, Hangzhou 310009, China
* Correspondence: lizhang@zstu.edu.cn (L.Z.); wangzi0209@zju.edu.cn (W.W.); shenjiawei@hznu.edu.cn (J.-W.S.); Tel: +86-571-2886-3425 (J.-W.S.)
† These authors contributed equally to this work.

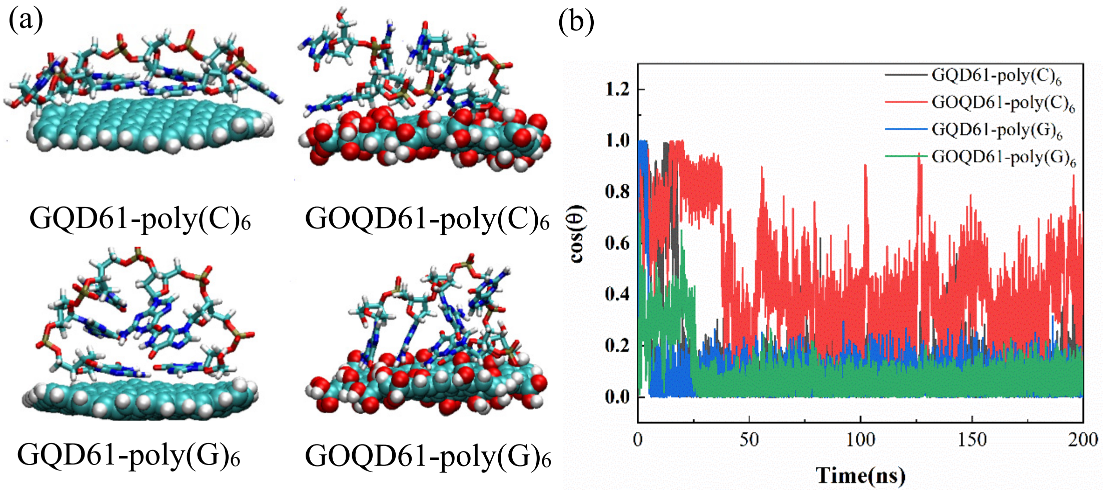

Figure S1. (a)The snapshot of ssDNA adsorbed on the surface of GQD61 and GOQD61 at the end of 200 ns MD simulation; (b)The angle between ssDNA axis and the normal of the GQD61 (GOQD61) surface as a function of simulation time.

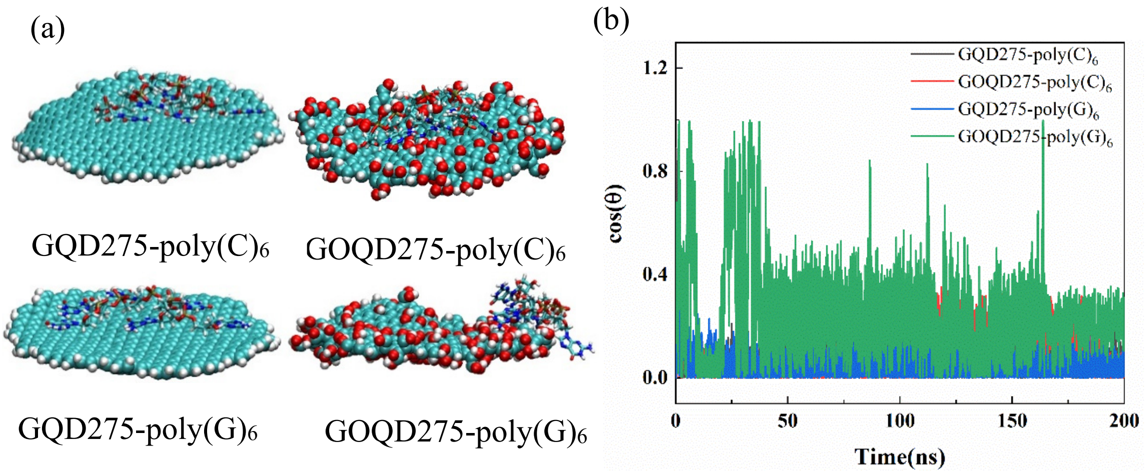

Figure S2. (a) The snapshot of ssDNA adsorbed on the surface of GQD275/GOQD275 at the end of 200 ns MD simulation; (b)The angle between ssDNA axis and the normal of GQD275/GOQD275 surface as a function of simulation time.

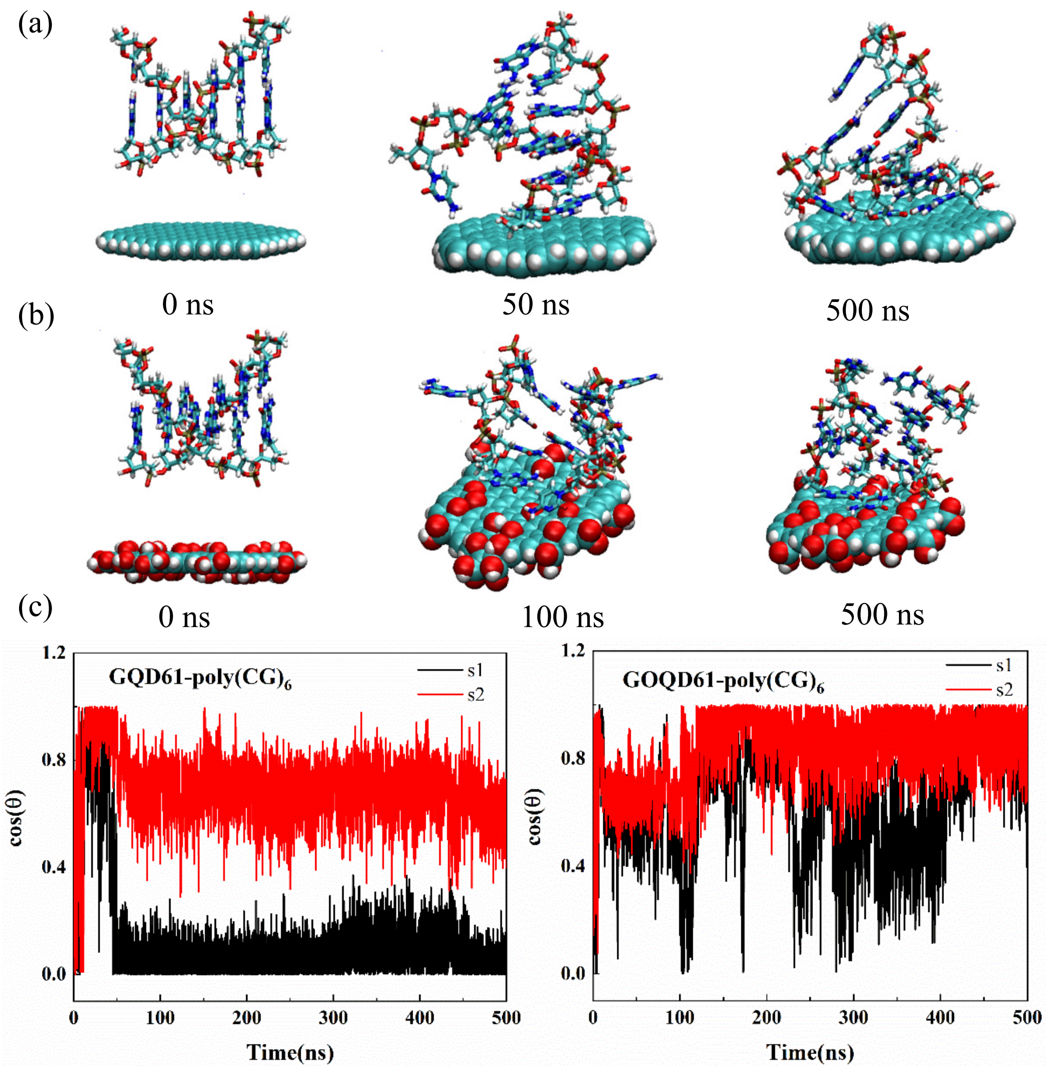

Figure S3. (a) The snapshot of dsDNA adsorbed on the surface of GQD61; (b) The snapshot of dsDNA adsorbed on the surface of GOQD61; (c) The angle between the axis of two single strands of dsDNA and the normal of the GQD61/GOQD61 surface as a function of simulation time.

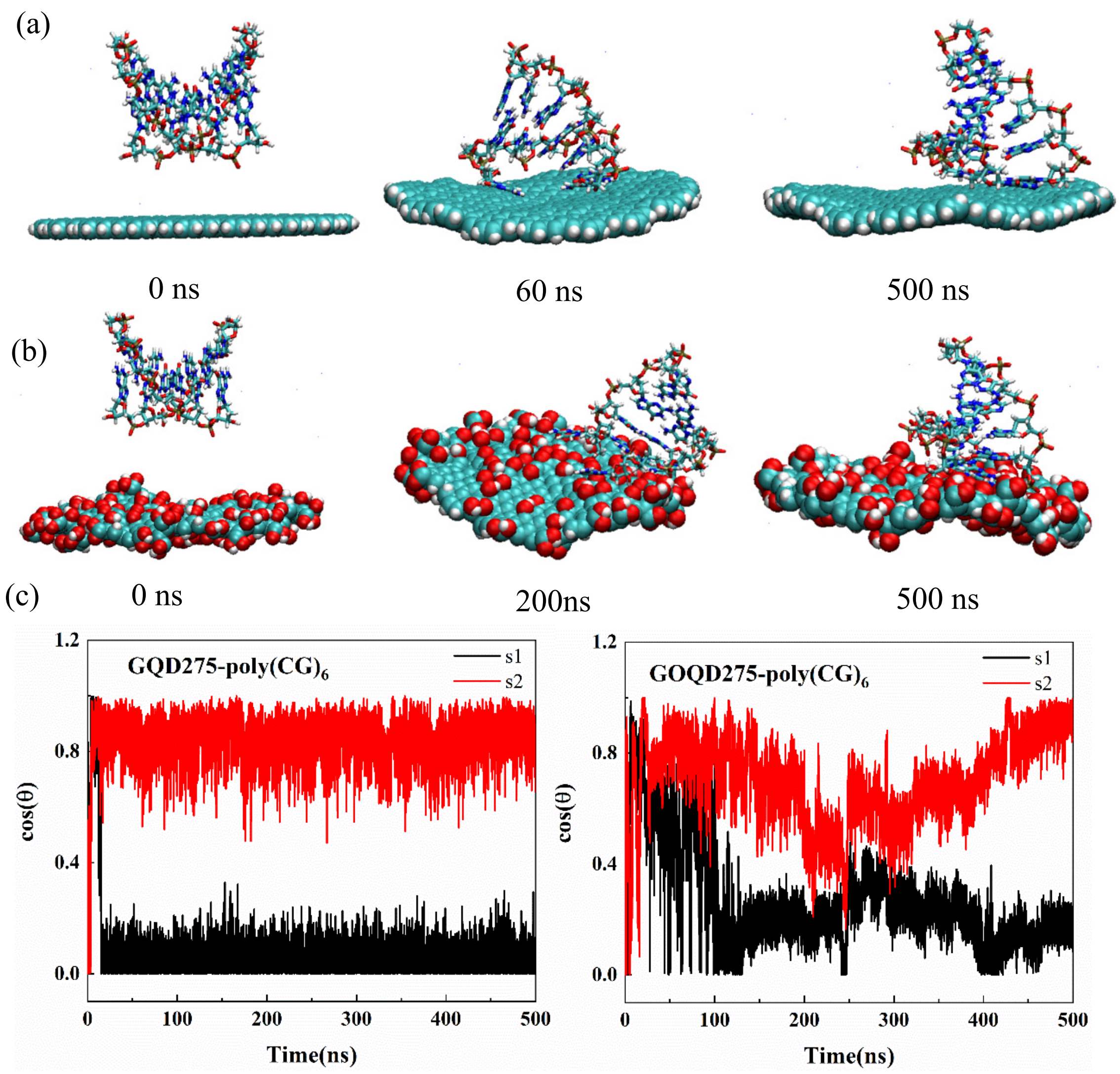

Figure S4. (a) The snapshot of dsDNA adsorbed on the surface of GQD275; (b) The snapshot of dsDNA adsorbed on the surface of GOQD275; (c) The angle between the axis of two single strands of dsDNA and the normal of the GQD275/GOQD275 surface as a function of simulation time.

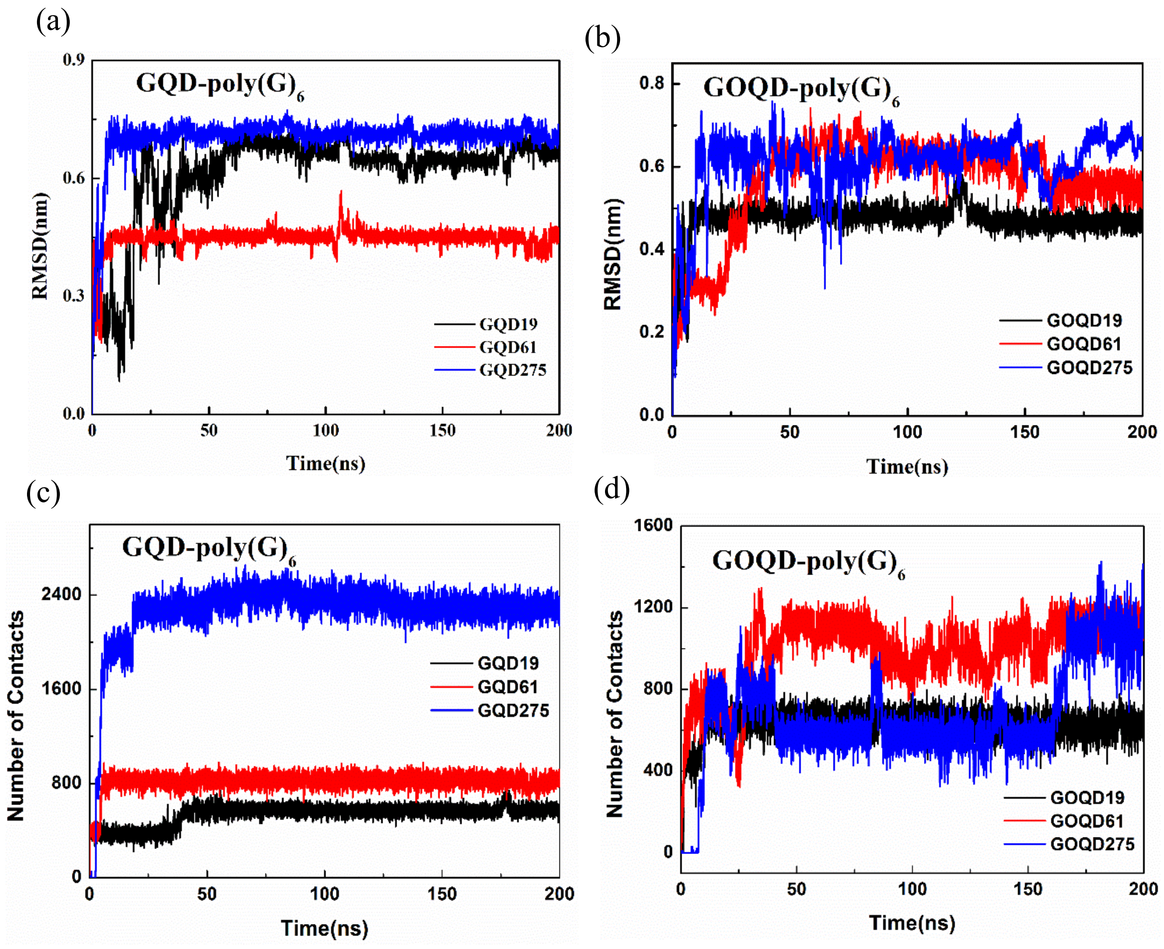

Figure S5. (a) The RMSD variation of poly(G)$_6$ during the adsorption process on GQD with different sizes; (b) The RMSD variation of poly(G)$_6$ during the adsorption process on GOQD with different sizes; (c) Number of contacts between poly(G)$_6$ and GQD versus the simulation time; (d) Number of contacts between poly(G)$_6$ and GOQD versus the simulation time.

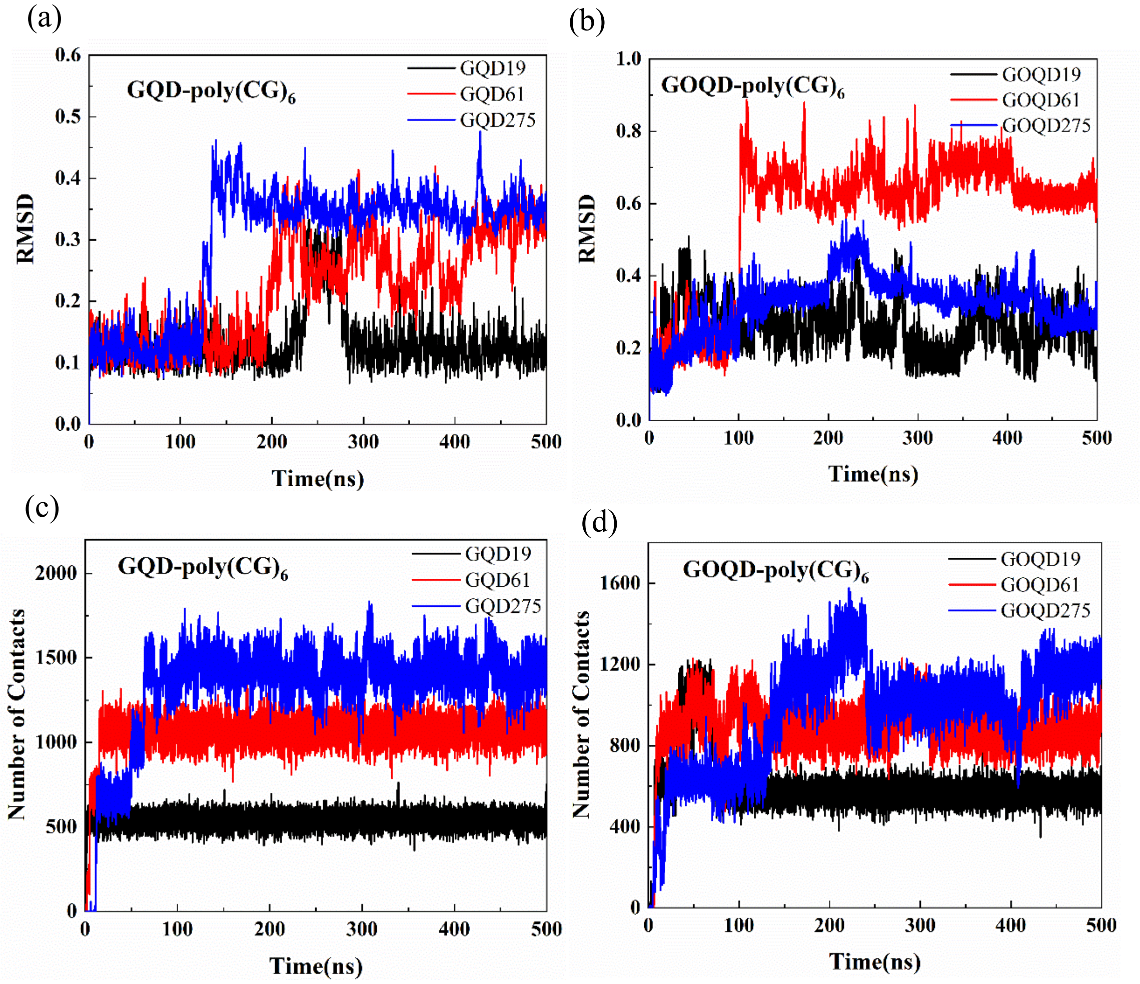

Figure S6. (a) The RMSD during the adsorption of dsDNA on the surface of GQD; (b) The RMSD during the adsorption of dsDNA on the surface of GOQD; (c) Number of contacts between dsDNA and GQD versus the simulation time; (d) Number of contact between dsDNA and GOQD versus the simulation time.
